# Embryonic exposure to predation risk and hatch time variation in fathead minnows

**DOI:** 10.1371/journal.pone.0255961

**Published:** 2021-08-12

**Authors:** Marianna E. Horn, Douglas P. Chivers

**Affiliations:** Department of Biology, University of Saskatchewan, Saskatoon, SK, Canada; Laboratoire de Biologie du Développement de Villefranche-sur-Mer, FRANCE

## Abstract

Organisms are exposed to a wealth of chemical information during their development. Some of these chemical cues indicate present or future dangers, such as the presence of predators that feed on either the developing embryos or their nearby parents. Organisms may use this information to modify their morphology or life-history, including hatching timing, or may retain information about risk until it gains relevance. Previous research has shown predation-induced alterations in hatching among embryonic minnows that were exposed to mechanical-injury-released alarm cues from conspecific embryos. Here, we test whether minnows likewise hatch early in response to alarm cues from injured adult conspecifics. We know that embryonic minnows can detect adult alarm cues and use them to facilitate learned recognition of predators; however, it is unknown whether these adult alarm cues will also induce a change in hatching time. Early hatching may allow animals to rapidly disperse away from potential predators, but late hatching may allow animals to grow and develop structures that allow them to effectively escape when they do hatch. Here, we found here that unlike embryonic fathead minnows (*Pimephales promelas*) exposed to embryonic cues, embryonic minnows exposed to adult alarm cues do not exhibit early hatching. The ability of embryos to recognize adult alarm cues as a future threat, but not a current one, demonstrates sophisticated ontogenetic specificity in the hatching response of embryonic minnows.

## Introduction

Animals facing predation must continually weigh the gains of activities such as foraging and mating against the risk of being injured or killed by a predator. Predation risk has a large impact on behaviour, morphology and life-history. The behavioural impacts are well studied; animals typically exhibit fright behaviours, such as reduced movement and foraging [1], and in the case of fish, increased shelter use and shoaling [2]. These behaviours may significantly decrease their risk of predation [3, 4]. Individuals that are under a constant threat of predation may even begin to demonstrate neophobia–a fear of all novel stimuli [5]. These more drastic behavioural responses may come at a greater cost, as exhibiting fear of everything may prevent low-risk gains–a willingness to take opportunities when the threat is low rather than non-existent [1]. Individuals that experience extended exposure to predation risk at certain life stages may also change morphologically to decrease their risk of predation. A classic example is the crucian carp, whose body shape deepens in the presence of pike predation [6]. This morphology is advantageous in the presence of the gape-limited pike, which cannot open their mouths wide enough to consume the deeper-bodied morphotype. When the predation risk is great, the cost of this morphological variation, specifically decreased swimming speed, is negligible compared to the decreased risk of predation [6]. Fathead minnows (*Pimephales promelas*) likewise change their morphology in response to risk, but this trait is restricted to males. Females appear to have a consistent morphology, whereas males exhibit considerable variation, with early alarm cue exposure inducing deeper head and body structures, as well as shorter caudal penduncles and fins and longer dorsal fins [7]. As morphological changes may help animals avoid predation, so too can life-history shifts such as time of hatch. Because some predators forage selectively on specific life stages of prey, either shifting stages early or late may help prey reduce their predation risk. For example, newly-hatched salamander larvae are at high risk of predation by flatworms. Sih and Moore [8] found that in the presence of flatworms, salamanders delayed hatching to postpone their encounters with these predators until they were larger and better able to withstand the attacks.

Newly-hatched organisms are commonly considered to be at their most vulnerable due to their size, their naivety and the sudden loss of their shell [9]. However, being an embryo is not without risk either, as its immobility makes it impossible for an embryo to escape danger. Risk-induced hatching variation is one of the few mechanisms by which an embryo can mitigate risk. Embryos typically hatch spontaneously at a certain stage under normal conditions, but may hatch prematurely or delay hatching in the presence of higher-than-normal cue levels or extreme conditions [10]. Threats such as low oxygen levels [11], pathogen presence [12, 13], elevated temperature [14, 15], risk of desiccation [16], high population densities or predation pressure [8, 17, 18] may all impact embryonic hatching times [10, 19]. In some cases the organisms can actually speed up their growth within the shell in order to hatch out earlier but at the same developmental stage, yet in other cases they simply escape their shell at an earlier developmental stage [20].

For some organisms that are under threat of predation, early hatching may afford them the ability to escape their predators. This phenomenon, has been observed across a wide range of taxa, including arthropods, amphibians and fish [17, 21–23]. Kusch and Chivers [17] showed that when fathead minnow embryos were subject to predation threat, as represented by repeated exposure to crayfish predation cues augmented by a single crushed embryonic conspecific, they increased the speed with which they hatched out of their shells, affording them the opportunity to seek shelter, while costing them developmental time within the egg. Individuals that hatched early due to predation pressure on the embryos had shorter fork lengths than their unthreatened conspecifics, the lifetime consequences of which are unstudied in fish. Studies in amphibians show a wide range of consequences, ranging from changes in hatching time, to morphological changes, to differences in growth rates, all varied by species [24]. Another study shows effects of predation-induced hatching that carry through two subsequent life stages in red-eyed tree frogs [23]. It is possible that while embryos that hatch early may be afforded the ability to escape whatever immediate predation pressure they incur, it may come at a significant cost over their lifespan, depending on what physiological changes are needed to induce early hatching.

The ability to respond to a threat is predicated on the ability to detect said threat. It has long been established that embryos are capable of identifying predation risk, usually via olfactory or mechanical cues. Mechanical cues may simply be direct physical contact. Chivers et al. [21] found that Pacific treefrogs (*Hyla regilla*) subject to direct mechanical contact from both predatory leeches (families Glossiphonidae and Erpobdellidae) and non-predatory worms hatched early. These responses were intensified by the combined presence of olfactory cues. Olfactory cues are often innately recognized cues from injured conspecifics in the form of alarm cues [2]. They may also come from innately recognized predators [25], but this is not typically the case in fish [26]. The cues could also theoretically even be learned predator cues, based on recent evidence that embryos can learn to identify predators in the embryonic stages [25, 27–29]. Interestingly, some fish are capable of discerning the ontogeny of the conspecifics that produced the alarm cues [30–32]. Fish that can differentiate between cues from differently aged conspecifics may have the benefit of being able to respond only to relevant cues, rather than responding to cues from a life stage that experiences different predation risk due to changes in size, habitat or behaviour.

In this experiment, we investigate whether alarm cues from adult minnows induce a change in hatching time in embryonic minnows and whether the concentration of the alarm cue impacts the hatch time.

## Methods

### Holding conditions

One hundred and twenty-five mating pairs of adult fathead minnows were obtained from Osage Catfisheries Inc. of Osage Beach, Missouri, USA. Each mating pair was placed in an individual 10-L glass tank (Hagen, Montreal, Canada) filled with dechlorinated water and an airstone. Fish were held on a light:dark cycle of 16:8 hours at 25.8°C (± 2.8). Because air stratification within the facility caused a temperature gradient of eight degrees (21–29°C), treatments were equally distributed across the shelves. A piece of polyvinyl chloride pipe cut in half was added to serve as a shelter for mating and egg-laying. Minnows were fed a combination of dried flakes and fresh brine shrimp. The adult minnows were removed from the tanks as soon as the eggs were deposited to prevent a parental care bias, as the father typically guards the eggs. The airstone was moved beneath the eggs to encourage oxygenation and reduce the risk of fungal infection. Any pair that spawned a particularly low viability set of eggs (less than ten percent hatch rate) was replaced with a different partner and allowed to have a second discrete mating event.

### Collection of cues

Four adult fathead minnows were sacrificed via a blow to the head (as per guidelines from the Canadian Council on Animal Care) for alarm cue collection. A thin layer of skin was removed from each side of each fish (1 cm^2^ per fish) and was homogenized in 40 ml of chilled, distilled water before being filtered through cotton batting to remove any remaining tissue. The solution was diluted to produce three concentrations of alarm cue stock: high, medium and low (1 cm^2^ of skin/ 40 L, 120 L or 240 L respectively). Thirty-ml aliquots of skin solution were stored at -20°C and thawed in a water bath prior to use. We know that these concentrations are sufficient to induce behavioural changes in minnows [33].

As these fish were going to be used for another experiment post-hatch, we also included a novel predator odor in four of our conditioning treatments. The predator odor is not expected to have impacted this experiment because many experiments with minnows have established that upon hatching minnows lack recognition of northern pike (*Esox lucius*) as a predator. However, for the sake of thoroughness, we explain the process of predator odor collection. Predator odor was collected from three pike (fork length 19.1 cm ± SE: 0.2) starved for one week prior to cue collection to prevent the presence of dietary alarm cues [34]. The pike were placed in individual 60 L glass collection tanks with only an airstone and clean dechlorinated water for 24 h to produce the predator odor. After the fish were returned to their regular tanks, the water was filtered through polywool. Bags of 125 ml of predator odor were stored at -20°C and thawed just before use.

### Conditioning

Starting 24–36 h after deposition, 138 clutches of eggs were exposed to the conditioning cues for one hour each morning and one hour each afternoon for two days. We did not believe there would be any gain to beginning conditioning prior to this time because the fundamental neural structure development would be incomplete (35). The egg surface (pipe) was transferred into a bucket with 1.5 L of water, which also contained one of 6 sets of conditioning cues: distilled water (25 mL distilled water); predator odor (20 mL predator odor + 5 mL DW); alarm cue mL high conspecific alarm cue control + 20 mL distilled water); Low (5 mL low concentration AC + 20 mL predator odor); Medium (5 mL medium concentration AC + 20 mL predator odor); High (5 mL high concentration AC + 20 mL predator odor). As mentioned, these conditioning treatments were designed for another experiment, but for our purpose they provide three concentrations of alarm cue, and a control to ensure that our addition of predator odor had no impact on hatching. The final concentrations of alarm cue in the treatment buckets were as follows: 1 cm^2^ / 73 200 L in the Low, 1 cm^2^ / 36 600 L in the Medium, and 1cm ^2^ / 12 200 L in the alarm cue and High, with none in the distilled water and predator odor buckets. After exposure, the pipe with the eggs was removed from the cue and placed in a clean water bath for two min before it was returned to its holding tank.

### Testing conditions

Eggs were checked every 4 h during the day (0600h, 1000h, 1400h, 1800h, 2200h) for signs of hatching. We considered clutches as replicates rather than individual eggs due to the large number of eggs (almost 17 000 in total). We noted the temperature at each 4 h interval, the times at which the eyes appeared, the first fish hatched, approximately 90% had hatched, and the last viable (not discolored) egg hatched, and recorded if there were any signs of fungal infection (common in eggs lacking parental care). Any infected eggs were carefully excised from the rest of the clutch to prevent pathogen spread.

### Statistical analyses

Because we were considering clutches rather than individual embryos, we used several different measures for hatching time, all recorded in hours. We used two starting points: time from deposition, and time from eye development, and three different end points: first hatch, 90% hatch, and final hatching of all viable eggs, for a total of 6 measures of hatching time. We included the time from eye development measure to prevent bias from our treatments not beginning until 24 hours (shortly before eye development at around 43 h). The appearance of eye spot pigmentation also lines up with developmental milestones including completion of fundamental structural neural development, including the telencephalon and olfactory bulb, which may be important for cue detection [35].

We used a correlation-based principal component analysis (PCA) to combine the six measures of hatching time into a single synthetic variable. We used this variable in an analysis of variance (ANOVA) to evaluate the effect of conditioning treatments with temperature as a covariate. No interaction was found between temperature and treatment (F_5,77_ = 1.330, p = 0.261). Clutches with hatching success rates of less than ten percent were excluded due to their low viability, leaving 118 clutches of eggs (subgroup of clutches with and without outliers, n = 18–22). We also looked at temperature across treatments to make sure there were not categorical differences.

### Animal welfare note

This study was conducted as approved by the University of Saskatchewan Committee on Animal Ethics under the protocol number 20070083. At the start of the experiment, four individuals were killed by a blow to the head to produce conspecific cues; MS-222 cannot be used as it interferes with cue production. Both the adults and the offspring were maintained for use for future experiments, but following their use in multiple experiments the study fish were euthanized with MS-222 as the permit did not allow release.

## Results

**Fig 1:**
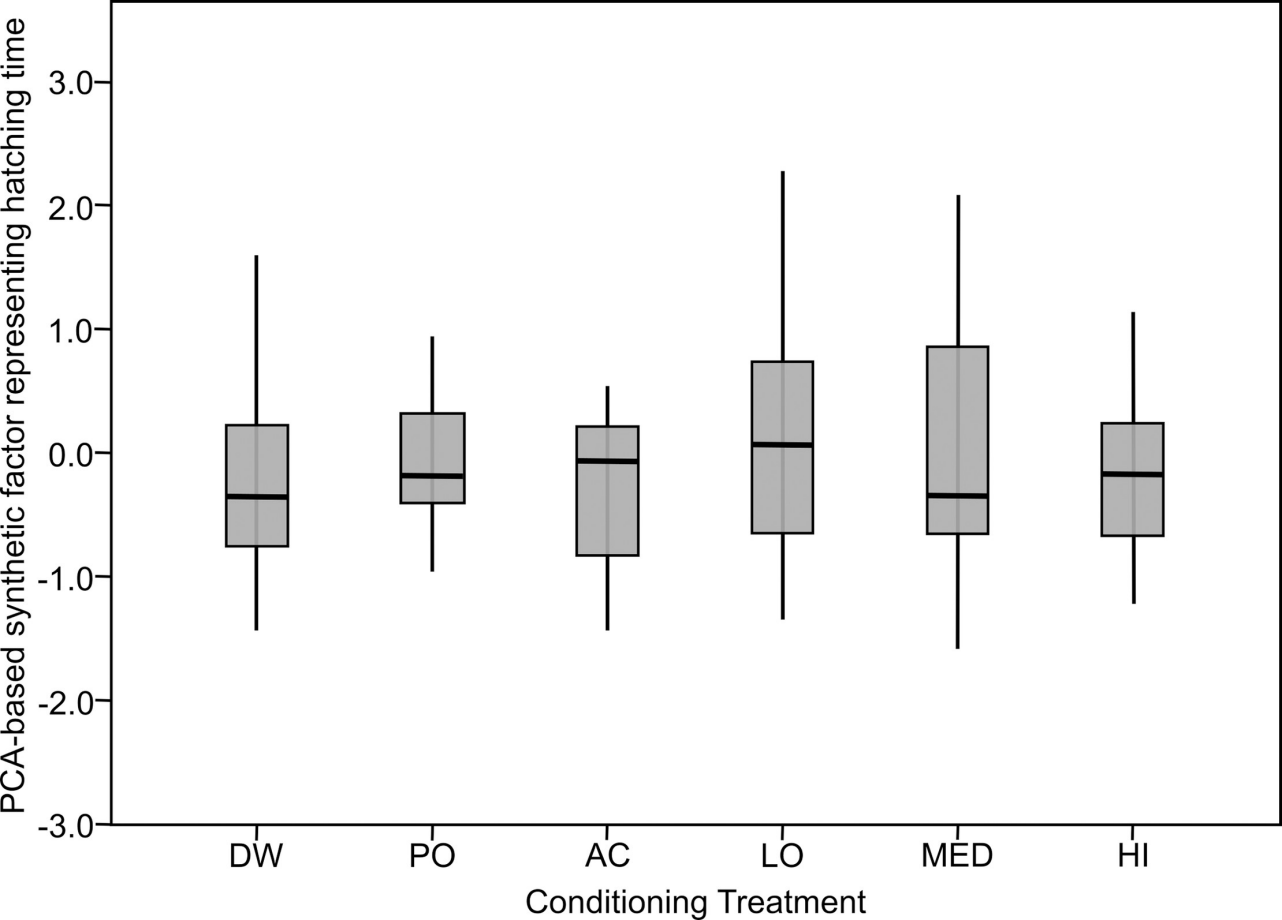
Variation in clutch hatching times following embryonic exposure to different treatment cues as represented by a PCA correlation-based synthetic factor that combines six measures of hatching time. DW = water, PO = predator odor, AC = alarm cue, LO = PO + low concentration AC, MED = PO + medium concentration AC, HI = PO + high concentration AC.

Clutch hatch rates (mean ± SE) from egg deposition ranged from first hatch at 79 h ± 1.7, to 90% hatch at 111 h ± 1.8, to final hatch at 122 h ± 1.8. From the appearance of eye spots the first hatch was at 35 h ± 1.3, 90% hatch at 67 h ± 1.2, and final hatch at 79 h ± 1.3. All data is available in the Supporting File (S1 Dataset). The first eigenvector of the PCA captured 73% of the variance and had correlation coefficients with the original response variables that ranged from 0.63 to 0.92. Clutch hatch rate was unaffected by treatment (F_5,110_ = 0.927, p = 0.467, Fig 1), but was affected by temperature (F_9,110_ = 1.330, p < 0.001). Temperature did not vary significantly across treatments (F_5,119_ = 0.196, p = 0.963).

## Discussion

Our results provide strong evidence that hatching rates were not affected by the presence of adult alarm cues. Given our design, unequivocal results and large sample sizes, we are confident that our findings are robust and reflect a true non-significant effect of risk on hatching time. Studies examining predator-induced hatching responses among fishes are at their infancy. We feel that it is just as important to publish results from carefully designed and executed studies that fail to find effects, as it is to publish those that confirm effects. The only significant decreases in hatching time observed in our experiment occurred as a result of increased temperature, which is a known phenomenon across various species of fish [36].

As mentioned, a myriad of factors can affect the hatching time of embryos. It may therefore seem inconsequential to have examined a factor that does not induce an effect. However, what makes this observation interesting is the fact that embryonic minnows seem to be differentiating between this and other highly similar pieces of information. Kusch and Chivers [17], for example, showed earlier hatching in minnows exposed to alarm cues from crushed conspecific embryos combined with feeding cues from virile crayfish fed embryos. In our experiment, we tested whether embryos exposed to adult alarm cues might also hatch prematurely, but found that they did not. The concentration of alarm cues from embryos and adults cannot be directly compared as it is measured by the numbers of eggs or the area (# of cm^2^ of skin) per volume (respectively). However, we do know from an experiment that followed from our current investigation, that the concentration of adult cues to which the embryos were subjected was enough to elicit a learned antipredator response [37], which clearly indicates that the concentration was adequate to indicate a threat. Despite the recognized threat to adults, however, these embryos did not hatch early. Although it is theoretically possible that ability to hatch early is not present in this genetic line of fathead minnows, as it was in the minnows used by Kusch and Chivers [17], many studies have demonstrated condition-dependent hatching rates across genetically independent fathead minnow populations [17, 36, 38, 39]. We believe it is far more plausible that the minnow embryos are responding differently to cues based on the ontogenetic stage of the donors.

Werner [40] suggests that shifts in the timing of life-history switch points, such as hatching or metamorphosis, should only occur in instances when the mortality/growth ratio of the current life stage is greater than that of the subsequent stage. Warkentin [18] expands this idea to propose that if embryos are in danger but juveniles are safe, early hatching would be favoured, and conversely, high juvenile mortality and safe embryos would favour a delay in hatching. The findings of Kusch and Chivers [17] follow this trend, with high embryonic risk inducing early hatching. Our experiment tests the reverse–a situation where embryos are not at risk, but adults are. We did not observe early hatching in this scenario, but neither did we observe delayed hatching. Nevertheless, when considered alongside Kusch and Chivers’ [17] work demonstrating early hatching in embryos following exposure to embryonic cues, our results suggest that the minnows are able to discern the ontogeny of the cues and use the information accordingly. Several species of fish are known to discern the ontogeny of conspecific alarm cues and to put more value in the cues from individuals of the same age. For example, Lönnstedt & McCormick [30] show a clear trend in the response of newly hatched damselfish to alarm cues of different aged conspecifics: response weakens as the age difference increases. Responses range from strong threat-sensitive responses to ontogenetically similar recruit-aged fish, to lower and non-threat dependent responses to juvenile cues, down to a complete lack of response to adult cues. Our findings indicate that minnows perceive the presence of a threat to a different life-stage (their free-swimming conspecifics) and recognize that this does not indicate a current threat to their safety. Because hatching early would not help them avoid this threat, they have no cause to hatch early. Indeed, in this instance, early hatching in the presence of a predator to adults would prove to be mismatched as it would increase their risk of mortality rather than decreasing it, not only through increased predator exposure, but also through the long-term costs associated with hatching at a less developed stage [23]. A sophisticated level of perception of risk should provide advantages to the current life stage by not inducing ill-advised premature hatching, while still allowing collection of information regarding future predation threats.

## Supporting information

S1 Dataset (XLSX)
